# Determinants of improved data consistency across routine immunization data tools for health facilities in Kano State, Nigeria

**DOI:** 10.11604/pamj.2020.36.148.20498

**Published:** 2020-07-02

**Authors:** Adekunle Akerele, Ramatu Obansa, Oluwasegun Joel Adegoke, Suleiman Haladu, Olorunsogo Bidemi Adeoye, Nnamdi Usifoh, Sulaiman Etamesor, Belinda Uba, Ndadilnasiya Endie Waziri

**Affiliations:** 1 African Field Epidemiology Network, Abuja, Nigeria,; 2 National Primary Health Care Development Agency, Abuja, Nigeria

**Keywords:** Supportive supervision, routine immunization, data consistency

## Abstract

**Introduction:**

in this study, determinants of improved data consistency for routine immunization information at health facilities was measured to identify associated factors.

**Methods:**

between June and August 2015, 1055 HFs were visited across 44 Local Government Areas in Kano state. We assessed data consistency, frequency of supportive supervision visits, availability of trained staff and attendance to monthly LGA RI review meetings. We compared RI monthly summary forms (MSF) versus national health management information system summary form (NHMIS) and vaccine management form 1a (VM1a) versus HF vaccine utilization summary monthly summary (HFVUM) for consistency. Data consistency at HF was determined at <+10% between number of children reportedly immunized, and doses of vaccine opened using 3 antigens (BCG, Penta and Measles). Levels of discrepancy <10% were considered as good data consistency. Bivariate and multivariate analysis used to determine association.

**Results:**

data Consistency was observed in 195 (18.5%) HFs between (MSF vs NHMIS) and 90 (8.5%) HFs between (VM1a vs HFVUM). Consistency between MSF vs NHMIS was associated with receiving one or more SS visits in the previous month (p=0.001), data collection tools availability (p=0.001), recent attendance to monthly LGA RI review meeting and availability of trained staff. Data consistency between VM1a form and the HF VU summary was associated with a recent documented SS visit (p=0.05) and availability of trained staff (p=0.05).

**Conclusion:**

low level of data consistency was observed in Kano. Enhanced SS visits and availability of trained staff are associated with improved data quality.

## Introduction

Routine immunization (RI) is a key pillar of the Global Polio Eradication Initiative (GPEI) program [1], designed to free the world of polio. In the African region, the transmission of poliovirus is limited to restricted areas with low community immunity and poor RI coverage. Nigeria is the only country in Africa that is yet to be certified as polio free with the last WPV1 case reported in September 2016. Weak routine immunization (RI) program and poor data quality contributes to the delay in freeing Nigeria of polio. Reliable and accurate public health information is essential for monitoring, evaluating and improving the delivery of health-care services and programs [2]. Studies of public health information systems in developing countries frequently document problems with data quality, such as incomplete records and untimely reporting. Gaps in the quality and accuracy of the reported routine immunization administrative data have been documented studies previously conducted [3]. Yet this system is the only data source available for the continuous, routine monitoring of health programs [2]. Identifying the challenges that cause health information systems to fail in delivery of quality information is a necessary first step to reverse these inadequacies. RI data from health facilities (HF) are the primary source used by health information systems to estimate administrative immunization coverage rates. In Nigeria, immunization services data collection and reporting are a part of a centralized large data collection system known as the as the National Health Management Information System (NHMIS) framework. Immunization Focal Persons (IFPs) at HFs compile vaccination data daily after each immunization session from the tally sheets and report these data to a Local Government Area or LGA (district) Immunization Officer (LIO) every month. The LIO collates these data and forwards them monthly to the state level and, finally, to the national level [3].

Much effort had been invested into improving the quality of data in the NHMIS including designing the District Health Information System version 2 (DHIS2) database as the hub for all NHMIS data in Nigeria [4,5]. The Nigerian DHIS2 platform has integrated an RI module to support a dedicated RI dashboard, providing access to real time data required to measure performance at the HF, LGA, state and national levels. The purpose of implementation of the DHIS-2 RI module in Nigeria was to improve data quality, facilitate data use for action and enhance and better target supportive supervision visits to low performing LGA and HF. The DHIS2 RI module and dashboard was implemented in Kano state between November 2014 and March 2017. To monitor and improve the RI data collected on DHIS2, the National Primary Health Care Development Agency (NPHCDA) introduced data quality supportive supervision (DQSS) which is an enhanced capacity building methodology designed to improve data generation through on-the-job training for health workers. This enhanced supportive supervision (SS) approach was built on existing structures for data quality improvement by providing an opportunity for more thorough review of RI data within all health facilities rather than just a small sample. The activity was conducted as part of the RISS structure to improve data quality at the HF level in Kano State. The purpose of this paper is to determine factors that are associated with improved data consistency at the HF level in Kano State, Nigeria.

## Methods

**Type of study:** this study was a cross-sectional assessment conducted between June and August 2015 in Kano state Nigeria.

**Study population:** the study population comprised of the HF-in-charge and RI focal person across all HFs providing RI services in Kano state.

**Sampling:** all 1160 HFs providing RI services across the 44 local government areas (LGA) in Kano state as obtained from the state primary health care management board were visited during the 3-month period between June-August 2015.

**Study design:** data quality supportive supervision (DQSS) was implemented as part of the HF assessment using the existing SS structure in Kano state. It was conducted by the LGA team consisting of the local immunization officers (LIO), the LGA monitoring and evaluation officers (M&E), the cold chain officers (CCO) and consultants from partner organizations. Using study protocols, supervisors conduct data quality checks during one of the planned SS visits. During the session, data tools are reviewed, health workers were provided with refresher training on the appropriate way of filling the tools to generate quality data. Three phases of training were conducted, (National, State/Zonal and LGA training) to guide supervisors on identifying reasons for inconsistency in data during the visits and administering the checklist developed for data extraction. The state/zonal and the LGA training took place in Kano. Participants at the state/zonal training included the key officials supporting RI interventions while the LGA level training, participants included LIO, M&E, CCO and consultants from partner organizations.

**Data collection:** the checklist was developed to collect the following information during the visit: Identifying information, number of doses of vaccines administered, number of doses of vaccine and devices utilized, data tools availability and storage, and whether supportive supervision was conducted to the HF recently. The checklist was validated by conducting visits to 4 LGAs and 12 HF between December 2014 and March 2015.

**Data analyses:** analysis was conducted using SPSS version 20. The variables analyzed include characteristics of the HF, pooled reported number of specific vaccines (BCG, Penta3 and Measles) given to children between June, July, August, pooled reported number vials of the same key vaccines opened in June, July, August, availability and management of RI data tools, documentation of recent supportive supervisory visits to the health facilities, and recent attendance at monthly review meetings. Univariate analysis was conducted on each variable to determine frequencies. Bivariate analysis was conducted to test for association between data consistency, and independent variables such as data tool availability, recent supportive supervisory visits, and recent attendance at monthly review meetings. Multivariate analysis was conducted to determine strength of the association using adjusted odds ratio as a measure.

**Calculating consistency of data reported:** we assessed data consistency over a 3 month period between HF monthly summary form (MSF) vs national health management information systems monthly summary form (NHMIS) and between vaccine management form 1a (VM1a) and health facility vaccine utilization summary (HFVUS). These documents are an integral part of the DQSS visits at the health facility level. We used a scatter plot from pooled data across the three months to display disparity across (MSF vs NHMIS) and (VM1a vs HFVUS). A discrepancy level of ≤10% between the forms to allow for minor errors was used to dichotomize the data consistency variable between (MSF vs NHMIS) and (VM1a vs VUS). Consistency was measured by using 3 specific antigens (BCG, Penta and Measles). HFs with discrepancy levels ≤10% are categorized as good consistency levels while those discrepancy >10% are categorized as poor consistency.

**Study approval:** This study was approved by the national primary health care development agency (NPHCDA) and the Kano state primary health care management board (KSPHCMB).

## Results

**Characteristics of health facilities:** we analyzed a total of 1055 checklists, one per HF visited of which 1035 (98.1%) were public. Among all the reporting HFs, 959 (90.9%), had at least one staff trained on the proper use of RI data tools while 230 (21.8%) had at least two staff trained. Among HFs, 926 (87.8%) reported that at least one staff attended LGA monthly review meeting in the last one month before the visit, 903 (85.6%) received a supportive supervision visit within one month before the visit and 840 (79.6%) had all RI data tools available at the time of visit.

**Figure 1:**
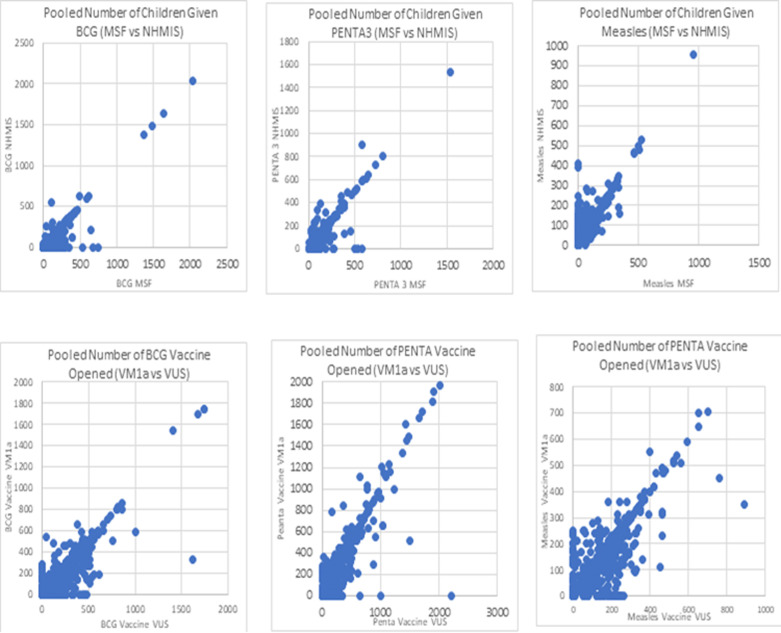
scatter plot showing disparity in records

**Data discrepancy:** in Figure 1, we presented scatter plots showing discrepancy between doses of antigens given to children has recorded on HF MSF and NHMIS MSF using 3 antigens BCG, PENTA3 and measles. Similarly, discrepancy between doses of antigens opened recorded on VM1A and HFVUS were also presented using 3 antigens BCG, PENTA and measles. Points were scattered more in vaccine doses opened recorded compared to recorded doses of children immunized.

**Assessing data consistency:** number of HFs with good data consistency in number of doses of specific antigens given to children recorded on HF MSF and NHMIS MSF for a 3-month period were PENTA3 (32.5%), BCG (28.7%) and measles (28.7%). Overall, 18.5% of health facilities had good data consistency in number of doses giving to children for all three of the selected antigens. Good data consistency was observed between number of doses opened recorded on VM1A and HFVUS over a 3 month period for PENTA vaccine (13.2%), BCG vaccine (31.7%) and measles vaccine (34.6%) in health facilities visited while overall good data consistency of vaccine doses opened across the three antigens was observed in 8.5% health facilities.

**Correlates of good data consistency:** good data consistency between the HF MSF and the NHMIS MSF was observed in 149 (76.4%) HF with at least one health worker trained on data tools, in 169 (86.7%), that have had all RI data tools available, in 180 (92.3%) where at least one staff attended the LGA review meeting at least one month before the visit and in 183 (93.8%) HF that had supportive supervision visit one month or less before the visit. Among HF with poor data consistency, 584 (67.9%) have at least one health worker trained on data tools, 671(78%) had complete set of data tools, 746 (86.7%) had attended LGA monthly review meeting less than one month before the visit while 720 (83.7%) had supportive supervision visit one month or less (Table 1). Furthermore, good data consistency between the VM1a and the HF vaccine utilization MSF across all three antigens (BCG, Penta 3 and Measles) was observed in 82 (91.1%) of HFs that had a SS visit at least one month or less before the visit, 85(94.4%) of health facilities with one person attending the LGA review meeting less than one month before the visit, in 78 (86.7%) of health facilities with complete routine immunization data tools, 70 (77.8%) of health facilities with at least one health worker trained on RI data tools. In HF with poor data consistency, 821 (85.1) had supportive supervision visit one month or less before the study, 841 (87.2%) had someone attend the LGA review meeting one month before the study, 762 (79.0%) had complete RI data tools and 663 (68.7%) had one staff trained on RI data tools. Availability of a trained health worker was significantly associated with data consistency between VM1A and HF vaccine utilization MSF (Table 2).

**Table 1:** consistency between HF MSF and NHMIS MSF versus characteristics of reporting HF

HF Characteristics	Categories	HF with Poor Consistency (N=860)	HF with Good Consistency (N=195)	Total (N=1055)	P-Value for Chi square test
Availability of trained staff at the facility	No	90 (10.5%)	6 (3.1%)	96(9.1%)	0.01
	Yes	770 (89.5%)	189 (96.9%)	959(90.9%)	
Number of Health Workers trained on RI tools per facility	No trained Health Worker	87 (10.1%)	5 (2.6%)	92(8.7%)	0.002
	One trained Health Worker	584 (67.9%)	149 (76.4%)	733 (69.5%)	
	At least 2 trained health	189 (22.0%)	41 (21.0%)	230 (21.8%)	
Tools Availability at the HF level	Incomplete RI data tools	189 (22.0%)	26 (13.3%)	215 (20.4%)	0.007
	Complete RI data tools	671 (78.0%)	169 (86.7%)	840 (79.6%)	
When was the last time someone from this HF attended LGA meeting	No Response	18 (2.1%)	5 (2.6%)	23 (2.2%)	0.015
	Less than 1 month	746 (86.7%)	180 (92.3%)	926 (87.8%)	
	2-4 months	21 (2.4%)	6 (3.1%)	27 (2.6%)	
	More than 4 months	75 (8.7%)	4 (2.1%)	79 (7.5%)	
When was the last time this HF had a RI SS visit from the LGA	No Response	89 (10.3%)	3 (1.5%)	92 (8.7%)	0.001
	One Month or less	720 (83.7%)	183 (93.8%)	903 (85.6%)	
	2-4 Months	29 (3.4%)	7 (3.6%)	36 (3.4%)	
	4 Months or more	22 (2.6%)	2 (1.0%)	24 (2.3%)	

HF with poor data consistency had >10% discrepancy level between HF monthly summary form (HF MSF) and NHMIS monthly summary form (NHMIS MSF) while HF with good data consistency had <10% discrepancy level between HF monthly summary and NHMIS monthly summary. Discrepancy was measured based on 3 antigens (BCG, PENTA 3 and Measles)

**Table 2:** consistency between VM1A and HF vaccine utilization MSF versus characteristics of reporting HF

HF Characteristics		HF with Poor Consistency (N=965)	HF with Good Consistency (N=90)	Total (N=1055)	P-value
Availability of trained staff at the facility	No	95 (9.8%)	1 (1.1%)	96 (9.1%)	0.006
	Yes	870 (90.2%)	89 (98.9%)	959 (90.9%)	
Number of Health Workers trained on RI tools per facility	No trained Health Worker	91 (9.4%)	1 (1.1%)	92 (8.7%)	0.023
	One trained Health Worker	663 (68.7%)	70 (77.8%)	733 (69.5%)	
	At least 2 trained health	211 (21.9%)	19 (21.1%)	230 (21.8%)	
Tools Availability at the HF level	Incomplete RI data tools	203 (21.0%)	12 (13.3%)	215 (20.4%)	0.083
	Complete RI data tools	762 (79.0%)	78 (86.7%)	840 (79.6%)	
When was the last time someone from this HF attended LGA meeting	No Response	79 (8.2%)	0 (0.0%)	79 (7.5%)	0.044
	Less than 1 month	841 (87.2%)	85 (94.4%)	926 (87.8%)	
	2-4 months	24 (2.5%)	3 (3.3%)	27 (2.6%)	
	More than 4 months	21 (2.2%)	2 (2.2%)	23 (2.2%)	
When was the last time this HF had a RI SS visit from the LGA	No Response	91 (9.4%)	1 (1.1%)	92 (8.7%)	0.053
	One Month or less	821 (85.1%)	82 (91.1%)	903 (85.6%)	
	2-4 Months	32 (3.3%)	4 (4.4%)	36 (3.4%)	
	4 Months or more	21 (2.2%)	3 (3.3%)	24 (2.3%)	

HF with poor data consistency had >10% discrepancy level between vaccine management tool 1A (VM1a) and HF vaccine utilization summary form while HF with good data consistency had <10% discrepancy level between vaccine management tool (VM1a) and HF vaccine utilization summary. Discrepancy was measured based on 3 antigens bacille Calmette-Guerin, Pentavalent and Meales vaccines

**Factors determining good data consistency:** among the correlates of good data consistency between health facility monthly summary form (HF MSF) and NHMIS monthly summary form, predictors included availability trained health workers on RI data tools (AOR=1.14, 95% CI=0.15 - 8.46), attendance at a monthly review meeting at least one month before the visit (AOR=1.94, 95% CI=0.24 - 15.7) and having a supportive supervisory visit at least once in one month or less before the visit (AOR=2.94, 95%CI = 0.067 - 12.89) (Table 3). Good data consistency is likely to improve when there are trained health workers within a health facility (AOR=1.20, 95%CI=0.70 - 2.039) (Table 3).

**Table 3:** multivariate model showing predictors of data consistency between HF MSF vs NHMIS MSF and VM1A vs HF vaccine utilization MSF

HF Characteristics	Categories	HF MSF and NHMIS MSF		VM1A and HF Vaccine Utilization MSF	
		Adjusted Odds Ratio	95% CI	Adjusted Odds Ratio	95% CI
Availability of trained staff at the facility	No^*^				
	Yes	1.14		0.21	0.00 - 21.05
Number of Health Workers trained on RI tools per facility	No trained Health Worker^*^				
	One trained Health Worker	0.66	0.07 - 6.65	1.28	0.01 - 131.53
	At least 2 trained health	1.22	0.83 - 1.79	1.2	0.70 - 2.04
Tools Availability at the HF level	Incomplete RI data tools^*^				
	Complete RI data tools	0.76	0.46 - 1.23	0.88	0.46 - 1.68
When was the last time someone from this HF attended LGA meeting	Non-Response^*^				
	Less than 1 month	1.94	0.24 - 15.68	0.19	0.02 - 2.39
	2-4 months	0.69	0.24 - 1.97	0.67	0.20 - 2.31
	More than 4 months	0.83	0.20 - 3.31	0.88	0.18 - 4.37
When was the last time this HF had a RI SS visit from the LGA	Non-Response^*^				
	One Month or less	0.28	0.02 - 3.29		
	2-4 Months	2.94	0.67 - 12.89		
	4 Months or more	2.78	0.51 - 15.07		

The table suggests the possible predictors of data consistency between HF monthly summary form (HF MSF) and NHMIS monthly summary form (NHMIS MSF). With none of the odds ratio significant, it is essential to be cautious in the interpretation of the results.

## Discussion

Good data consistency across routine immunization data tools is an evidence of improved quality of data generated at the HF. This study suggests that data consistency can be improved by having trained staff to complete RI data tools, frequent conduct of supportive supervisory visits, attendance of monthly LGA review meetings and availability of complete RI data tools. Because multivariate analysis did not provide statistically significant results for any one variable, these characteristics appear to be all collinear or associated with one another. Data consistency between HF MSF and NHMIS form was associated with availability of trained staff on data tools, availability of complete RI data tools, attendance of monthly LGA review meetings and when last supportive supervision was conducted. Previous experience shows that a data quality improvement intervention that involves specific training for health-care workers on the importance of public health information, as well as monthly data reviews, feedback, routine data audits can be effective in increasing the accuracy of the data used to monitor prevention of mother to child transmission (PMTCT) services. Findings from this study suggests the possibility of a similar result when applied to RI data collection and reporting processes in Nigeria. Findings from the study indicate that a low number of HFs have data consistency between HF MSF and the NHMIS MSF in the three months of data reviewed. Some of the reasons identified for low consistency included poor harmonization process, wrong calculations and loss of data due to delay updating of data tools. This validates what was reported in a data quality audit study conducted in Anambra where inaccurate data and a low-quality data reporting was observed in most HFs visited [4]. Based on this finding, it is essential to cautiously use this data in measuring immunization performance. The credibility of decisions made and the appropriateness of guidance that these data might provide may be questionable.

Health facilities that received supportive supervisory visits more frequently tend to have good data consistency. This agrees with other studies that suggest a strong HMIS is fundamental to good data reporting process within a health system [6,7]. Supportive supervisory visits provide an opportunity to address data quality issues by allowing supervisors to promptly identify inconsistencies that will need further attention and a plan for a follow up and resolution. Enhancing the quality of supportive supervisory visits through mentoring of HCW and conducting on-the-job training could provide an opportunity to improve health worker´s data management skills through on the job training and mentoring. If Kano state can demonstrate commitment to ensuring that health facilities are visited, and that data spot check can be included, an opportunity to improve the quality of data generated may be presented. Different approaches can be used to improve the support mechanisms, for example, improving the quality of the supervisory visits as well as providing adequate feedback mechanism to the producers of data at the remote sites

The correlation between availability of trained staff on routine immunization data tools in health facilities and good data consistency was also observed in the study. Ensuring that each of the health facilities have relevant staff with appropriate knowledge on filling data tools can help improve quality of data. In a study conducted in Kyrgyzstan, South Africa and Malawi, it was reported improved data quality by giving health workers the basic skills to monitor their own work, leading to a sense of ownership of the generated information [6]. In a study in Ethiopia, it was reported that the availability of trained staff was associated with improved data quality [8]. Encouraging staff to be continuously trained is a good way to ensure that data quality can be improved at the HF. The study also showed that the frequency of attending monthly review meeting was also associated with good data consistency. This finding reiterates the importance of ensuring that review meetings are held at all levels of data governance. Such meeting provides an opportunity for peer review of data and identification of causes of error as well as feedback to the health workers. The monthly review meetings are an integral component of the health information reporting process and are a valuable forum for establishing and maintaining good communication between levels of the health system. They provide an opportunity for staff to discuss performance achievements and challenges, with the goal being to improve program services. The Kano state government should continue to ensure that their staff conduct supportive supervision visit to health facilities and monthly review meetings are sustained as a means of enhancing data use for action at all levels. Taking ownership of the process with provision of appropriate funding for the SS visits and coordination of the data use activity would strengthen the process.

## Conclusion

To improve data consistency, monthly conduct of supportive supervisory visits, attendance of monthly review meetings and availability of trained RI staff in the health facility should be given priority in Kano state.

### What is known about this topic

- Routine immunization data generated from health facilities;
- Designated health workers fill data tools at the health facilities and submit monthly;
- Submitted data is entered into DHIS2 monthly.

### What this study adds

- Factors that affect records on children immunized and vaccines used differ;
- Predictors of improved data consistency across data tools provided;
- Odds of each factor improving data consistency identified.

## References

[1] Blanche-Philomene MA, Edna M, Teka A, Mable C T (2020). Contribution of polio eradication initiative to strengthening routine immunization: Lessons learnt in the WHO African region.

[2] Mphatswe W, Mate K, Bennett B, Ngidi H, Reddy J, Barker PM (2012). Improving public health information: a data quality intervention in KwaZulu-Natal, South Africa. Bull World Health Organ.

[3] World Health Organisation (2008). Health information systems. June.

[4] Fatiregun A (2013). Accuracy and Quality of Routine Immunisation Data Monitoring System in two South-Eastern Districts of Nigeria. Nigerian Health Journal.

[5] Daskalakis TG (1992). Improving data collection. NAHAM Manage J.

[6] Odhiambo-Otieno GW (2005). Evaluation of existing district health management information systems a case study of the district health systems in Kenya. Int J Med Inf.

[7] Makombe SD, Hochgesang M, Jahn A (2008). Assessing the quality of data aggregated by antiretroviral treatment clinics in Malawi. Bull World Health Organ.

[8] Teklegiorgis K, Tadesse K, Mirutse G (2016). Level of data quality from Health Management Information Systems in a resource limited setting and its associated factors, eastern Ethiopia. SA J Inf Manag.

